# Development and Evaluation of a Duo Chikungunya Virus Real-Time RT-PCR Assay Targeting Two Regions within the Genome

**DOI:** 10.3390/v11080755

**Published:** 2019-08-15

**Authors:** Laurence Thirion, Laura Pezzi, Iban Corcostegui, Audrey Dubot-Pérès, Alessandra Falchi, Xavier de Lamballerie, Remi N. Charrel

**Affiliations:** 1 Unité des Virus Emergents (UVE: Aix Marseille Univ., IRD 190, INSERM 1207, IHU Méditerranée Infection), 13005 Marseille, France; 2 EA7310, Laboratoire de Virologie, Université de Corse-Inserm, 94925 Corte, France; 3 Emerging Pathogens Institute, University of Florida, Gainesville, FL 32601, USA

**Keywords:** *Alphavirus*, *Togaviridae*, emergence, arbovirus, arthropod-borne, fever, epidemic, outbreak, tropical disease

## Abstract

Chikungunya virus (CHIKV) re-emerged as a globalized health threat fifteen years ago. There are dozens of RT-PCR assays published. An inventory of the latter was made, and after *in silico* analysis, two assays were selected for their ability to detect strains belonging to the five CHIKV genetic lineages. They were combined in order to provide a robust assay not affected by genetic point mutations and the resulting Duo CHIKV real-time RT-PCR assay was compared to the two parental single-plex tests against five strains belonging to the five genetic lineages. The Duo CHIKV assay performed equally, or better, in terms of sensitivity, specificity, linearity and signal intensity. Dual-target assays are better suited for viruses having the propensity to evolve into new variants via point mutations or major sequence deletions/insertions. Here, we demonstrated that combining two single systems into a dual-target assay did not impair sensitivity and specificity, and proved a potent diagnostic tool to face a potential emergence of CHIKV variants by newly evolving mutations.

## 1. Introduction

Fifteen years ago, Chikungunya virus (CHIKV) re-emerged as a global health threat because of its important human pathogenesis combined with its propensity for globalization. First identified in Tanzania in early 1952, it has caused periodic outbreaks in Asia and Africa since the 1960s [1]. The explosive outbreaks in the Indian Ocean, South-East Asia (2005–2007) and in the Americas (2013–2016) allowed the spread of the disease over a wider range, with more than five million cases over the last 15 years [2,3]. Recently, locally-acquired cases of CHIKV infection have been identified in Italy and France [4,5,6,7], proving that Europe has suitable conditions for the autochthonous transmission of CHIKV.

Chikungunya disease causes a dengue-like syndrome, characterized by fever, rash and arthralgia. This clinical picture does not allow to distinguish CHIKV infection from other pathogens causing similar illnesses (o’nyong-nyong virus, Mayaro virus, Dengue virus) based on clinical grounds [8]. Thus, laboratory identification of CHIKV infection has become an important issue for medical and epidemiological purposes. In particular, real-time PCR assays allow viral detection at the early stage of the infection, before the rise of IgM antibodies; they are the most frequently used technique used for routine diagnosis because of their sensitivity, specificity and short-turnaround time.

This study aimed at compiling a list of all RT-qPCR assays available in literature in order to select the best suited for the detection of CHIKV strains belonging to the five genetic lineages, and to attempt to combine two of these assays into one in order to provide a test that is not only at least equally sensitive to the two parental ones, but also to provide a test that is more robust when facing possible false negatives due to point mutations or other evolutionary events frequently encountered with RNA genome viruses. To do so, (i) we performed *in silico* analysis for mismatch detection, (ii) we selected the two best suited RT-qPCR systems targeting different regions of the genome, (iii) we evaluated a combined Duo RT-qPCR assay against the two parental assays to assess the sensitivity of the Duo assay compared with that of the two parental assays individually, and the specificity against a panel of arboviruses that either belong to the genus *Alphavirus* or can be encountered in CHIKV epidemic and endemic areas, and are to be considered in the panel of agents for differential diagnosis.

## 2. Materials and Methods

### 2.1. In Silico Analysis

RT-qPCR assays for the diagnosis of CHIKV were searched for in NCBI PubMED CHIKV using “chikungunya” and “polymerase chain reaction” or “PCR” until December 2018. A total of 25 complete sequences representative of the five CHIKV genotypes (Asian, West African, East-Central-South African 1–3) were selected and aligned using MEGA 7 software. Primers and probes described in the literature were plotted against sequence alignment and compared for the number and position of mismatches. The choice of the two CHIKV RT-qPCR primers and probe sets to include in the Duo CHIKV RT-qPCR assay is discussed in the paragraph “Inventory of CHIKV RT-qPCR Assays and Selection” of the Results section.

### 2.2. RT-qPCR

RT-qPCR reactions for the two reference assays and the “designed in this study” Duo RT-qPCR were performed with SuperScript^®^ III Platinum^®^ One-Step RT-qPCR Kit with ROX (#11732-088, Invitrogen-Thermo Fisher Scientific, Waltham, MA, USA) on a BioRad CFX96^TM^ thermal cycler, software version 3.1 (Bio-Rad Laboratories, Hercules, CA, USA). A 5 μL volume of RNA was added to 20 μL of mix containing 12.5 μL of 2X Reaction Mix, 0.5 μL of Superscript III RT/Platinum Taq Mix and primers and probe at the concentrations described in Table 1. Cycling conditions were: 50 °C for 30 min; 95 °C for 2 min; 45 cycles of 95 °C for 15 s and 60 °C for 45 s. All probes were labeled with the same dye (FAM). There are no modifications for neither the sequence nor the concentrations of the primers and probes. The only difference is that the quencher of the probe described by Panning et al. has been modified for TAMRA, instead of BHQ in the original article. The reason for this is the need to have the same quencher for the probes of the two assays included in the Duo test.

### 2.3. Generation of CHIKV RNA Synthetic Transcript

A house-made synthetic standard RNA was used for the evaluation process of Duo CHIKV RT-qPCR assay, targeting one of the monoplex RT-qPCR included in the Duo assay. The target region, included in a plasmid synthetized by Genscript (GenScript, Piscataway, NJ, USA), was amplified by PCR. A first purification was performed using Monarch PCR & DNA Cleanup Kit (New England BioLabs, Ipswich, MA, USA). The RNA transcript was synthetized in vitro by using MEGAshortscript^TM^ T7 Transcription Kit (Invitrogen-Thermo Fisher Scientific, Waltham, MA, USA) according to the manufacturer’s instructions. TURBO DNase included in the same kit was used to remove any residual DNA. The RNA transcript was purified using Monarch PCR & DNA Cleanup Kit (New England BioLabs, Ipswich, MA, USA). The RNA concentration was determined using a Thermo Scientific^TM^ NanoDrop^TM^ (Thermo Fisher Scientific, Waltham, MA, USA). The RNA transcript was serially diluted from 10^8^ to 10^2^ copies/µL, and dilutions were stored at −80 °C.

### 2.4. Sensitivity

The measure and comparison of the sensitivity of the two selected assays and of the Duo assay was done with five strains of CHIKV representing the five genetic lineages: Asian, West African, and the three Eastern Central South African (ECSA 1, ECSA2, ECSA3), listed in Table 2. Serial dilutions of the quantitated freeze-dried cell culture supernatant were prepared using AVE buffer containing 1 µg/mL of RNA carrier (QIAGEN, Venlo, The Netherlands), in order to achieve 5-fold serial dilutions containing 10^2^ to 8.75 × 10^5^ RNA copies/mL. Six decreasing concentrations were tested using six replicates for each. A Ct ≥ 40 was considered as negative. LOD was defined as the number of RNA copies/µL contained in the highest dilution for which all 6 replicates were positive.

### 2.5. Specificity

Pathogenic alphaviruses (*n* = 8), flaviviruses (*n* = 11) and phleboviruses (*n* = 3) were selected, and they are listed in Table 1. All the viral strains included in the specificity panel were provided by European Virus Archive Goes Global (EVAg, https://www.european-virus-archive.com/), except Ross River virus, provided by the National Collection of Pathogenic Viruses (NCPV, https://www.phe-culturecollections.org.uk/collections/ncpv.aspx).

## 3. Results

### 3.1. CHIKV RT-qPCR Assays and Selection

**Figure 1:**
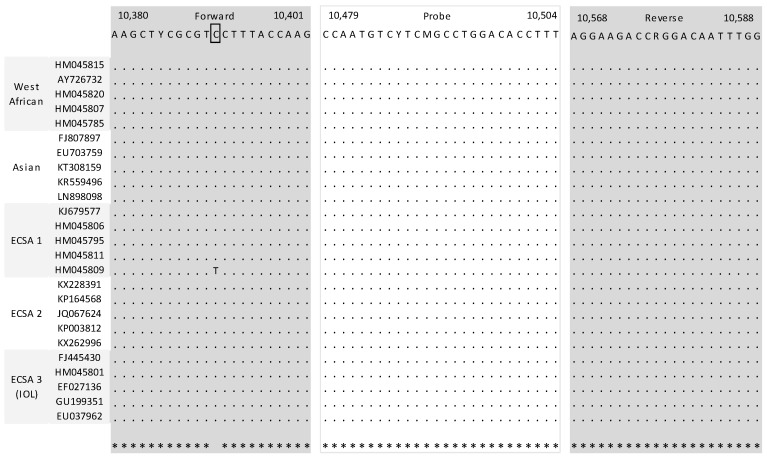
Mapping of primers and probe of selected systems described by Pastorino et al. Nucleotide positions refers to the sequence of Ross strain of CHIKV (ECSA1 lineage) (001v-EVA1455; GenBank accession number MG280943).

**Figure 2:**
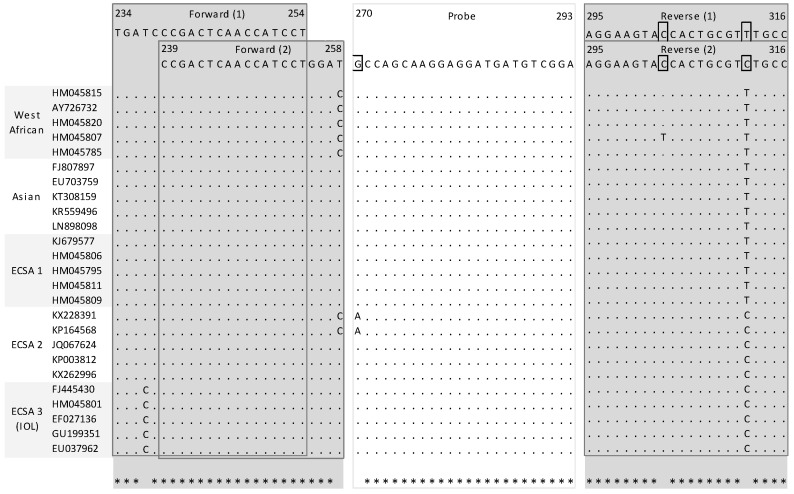
Mapping of primers and probe of selected systems described by Panning et al. Nucleotide positions refers to the sequence of Ross strain of CHIKV (ECSA1 lineage) (001v-EVA1455; GenBank accession number MG280943). (1) refers to primer sets common for all CHIKV lineages, (2) refers to the second set of primers that is targeting specifically strains belonging to the epidemic Indian Ocean lineage.

A total of seven assays were retrieved from the PubMED search [9,10,11,12,13,14,15]. Specific attention was given to mismatches located in the five nucleotides places at the 3′ extremity because of their ability to prevent correct hybridization. In probes, one or two mismatches generally do not compromise the hybridization potential but this also depends on the length of the probe and its G + C content. When compared with the 25 sequence alignments, two assays clearly provided a lower number of mismatches and mismatches that were not located in critical position. Detailed analysis had also been performed in a recent article, the leading author of which is one author of this study [8]. The first system, (Pastorino et al., 2005) designed to detect CHIKV strains belonging to both African and Asian lineages, consists of two primers and one probe which are located at the 3’ untranslated region [9]. Comparison with sequence alignment showed one possible mismatch within the forward primer with one strain belonging to the ECSA1 lineage at a position that is of little influence for hybridization (Figure 1). There was no mismatch within either the reverse primer or the probe. The second system, (Panning et al., 2006) consists of one fluorogenic probe and two sets of sense and reverse primers targeting the nsp1 gene: One set of primer is adapted to all CHIKV lineages, whereas the second one is best suited for strains belonging to the Indian Ocean lineage [10]. Few mismatches were observed in the in silico analysis, especially between the first couple of primers and ECSA3 strains sequences; however, the presence of a second set of primers complements the possible flaws of the first set (Figure 2).

### 3.2. Analytical Sensitivity of Duo CHIKV RT-qPCR Assay

The results are presented in Table 3. With no exception, the Duo assay showed equal or better sensitivity compared to the two single assays. For Asian and ECSA2 strains, the Duo assay provided 6/6 for 8 and 13 RNA copies/µL compared with 32 and 44 RNA copies/µL with the two mono-target assays, respectively. For West African, ECSA1 and ECSA3 strains, both Duo and mono-target assays detected 6/6 replicates for 24, 39 and 41 copies/µL, respectively. The Duo assay detected all strains with 6/6 scores for copies with the number varying from 8 to 41 per µL; the two single assays showed similar sensitivity with 6/6 scores for 24–44 RNA copies/µL.

### 3.3. Specificity of Duo CHIKV RT-qPCR Assay

The specificity of the assay was tested against several related and non-related viruses from *Alphavirus*, *Flavivirus* and *Phlebovirus* genera. The 22 strains incorporated in the panel correspond to viruses that are present in areas where CHIKV is circulating and to other alphaviruses distinct from CHIKV. Five CHIKV strains of different lineages were used as positive controls. Duo CHIKV RT-qPCR detected all CHIKV strains and did not react with the 22 viruses included in the exclusivity test panel, with a specificity of 100%.

### 3.4. Linearity of Duo CHIKV RT-qPCR vs. Pastorino and Panning Mono-Target Assays

**Figure 3:**
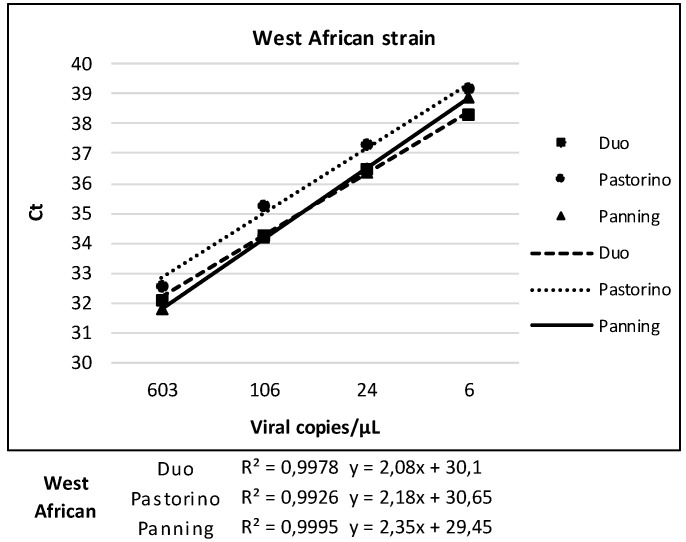

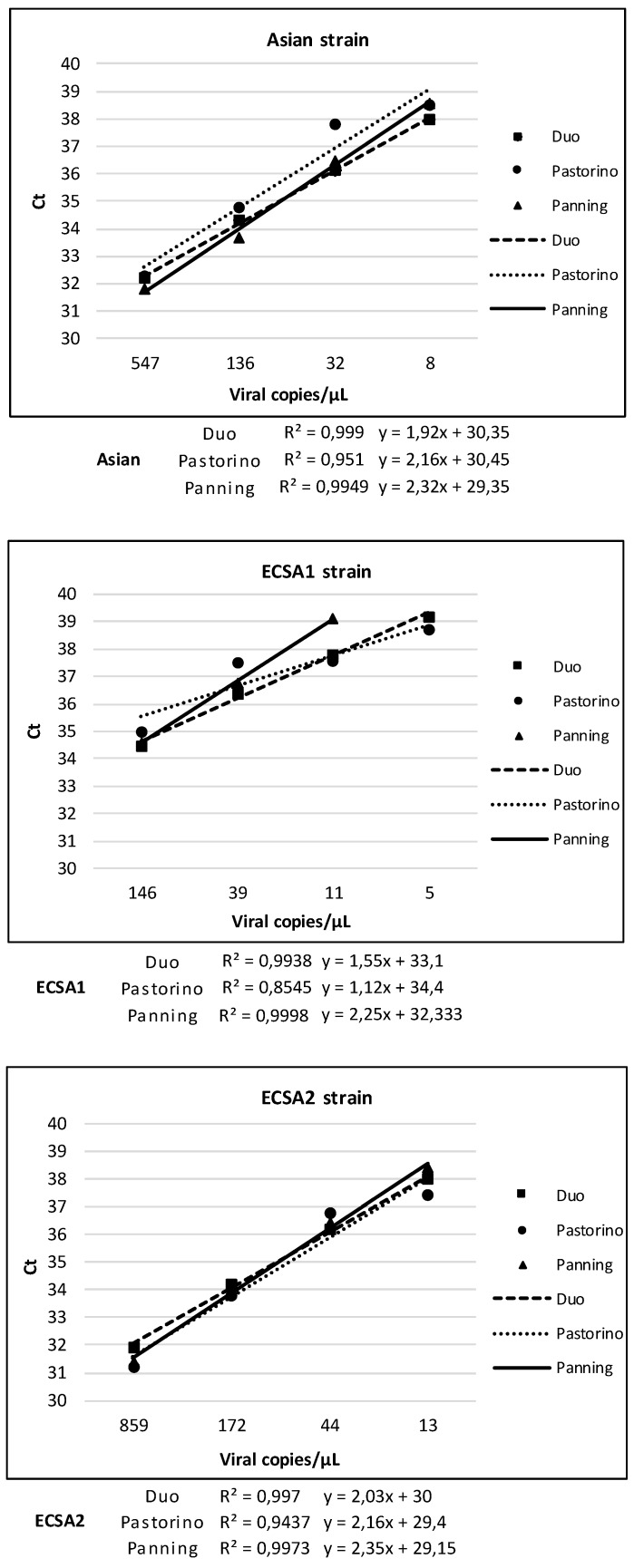

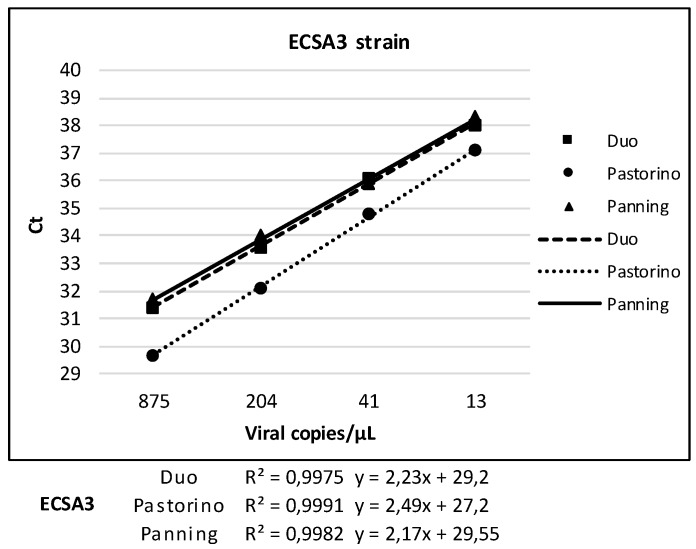
Linearity of Duo CHIKV RT-qPCR vs. Pastorino and Panning Mono-Target Assays.

The results about the analytical sensitivity test obtained with the Duo CHIKV RT-qPCR test were compared to results of the mono-target assays, Pastorino and Panning (Table 3), in order to evaluate the correlation between Ct values and the six different CHIKV concentrations previously tested. The response (Ct) for the three systems is linear, with correlation coefficient (R^2^) close to one for all CHIKV strains. Results are detailed in Figure 3.

### 3.5. Signal Intensity

**Figure 4:**
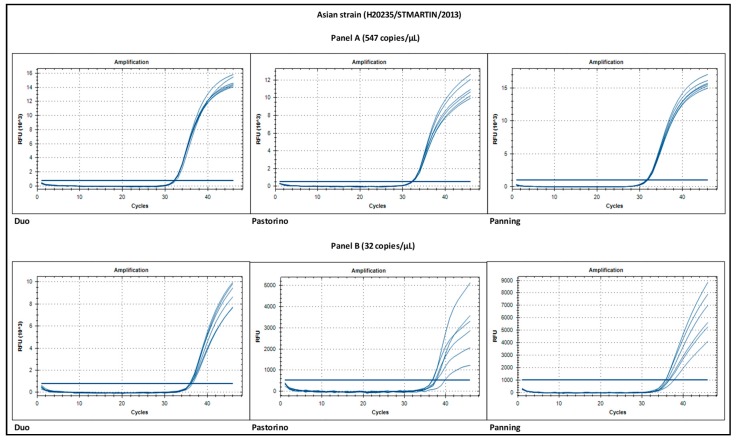
Intensity of fluorescence signal of CHIKV Asian strain at high (**A**) and low (**B**) RNA copy number.

Signal intensity is important for it has a significant impact on the ability to discriminate between negative and positive results without ambiguity. This situation is usually observed when the tested sample contains a low number of RNA copies, thus inducing a signal that is weak. As shown in Figure 4, low copy samples that are close to the limit of detection provide a much stronger signal with the Duo CHIKV RT-qPCR assay compared to mono-target assays.

## 4. Discussion

In this study, we developed and evaluated a Duo Chikungunya RT-qPCR assay; its dual-target design is characterized by the creation and detection of two different amplicons based on two different sets of primers and probes. Otherwise, we identified in literature several dual-target commercial tests for the screening of non-arboviral pathogens: Among them, Roche COBAS TaqMan HIV-1 assay (Roche Holding AG, Basel, Switzerland) and Hologic Aptima HIV-1 Assay (Hologic Inc, Marlborough, MA, USA) have been evaluated [16,17,18] and their use is recommended for blood donor screening test of HIV-1. In Germany, for example, from 1 January, 2015, the use of dual-target NATs is mandatory to screen cellular blood components, therapeutic individual plasma and stem cell preparations for hematopoietic re-constitution for HIV-1 [19]. This obligation was introduced after six cases of HIV-1 RNA-positive blood donations occurred between 2007 and 2012, despite routine NAT screening, of which two resulted in HIV transmission to the recipients by transfusion of the corresponding red blood cell (RBC) concentrates [20,21,22,23]. HIV-1 detection failures were due to mono-target assays measuring viral variants that exhibited mismatches with primer or probe sequences of the NAT target region.

The advantages of a dual-target design are multiple. Firstly, it allows us to detect viral genomes exhibiting mutations, as well as new viral variants. Polymorphisms, point mutations or major sequence deletions/insertions can compromise the accuracy of diagnostic and screening tests, resulting in false-negative NAT results despite viral RNA at concentration levels sufficiently high for detection by NAT. The inclusion of two targets into the assay design assures that the failure of one region detection is compensated by the detection of the other. This is of particular interest for CHIKV detection: Its potential to emerge and sustain its circulation in new environments relies on its ability to undergo genetic changes, in order to adapt to new hosts. In fact, the 2005–2006 epidemic in the Indian Ocean was associated with a strain of CHIKV with a mutation in the envelope protein gene (E1-A226V) [24,25]. Probability of CHIKV detection, compensating for mutations or mismatches, can be increased by the choice of two, well-conserved genome regions to be targeted by NAT tests.

Secondly, a dual-target design allows an easy interpretation of the results. The use of a unique fluorescent dye for both probes (as we chose for our Duo CHIKV RT-qPCR assay) provides a simple algorithm for the validation of the analytical results. A negative result corresponds to the lack of amplification by both assays; a positive result corresponds to the detection of a viral genome by either both assays or one assay only, without the possibility to distinguish the two situations, avoiding endless use of confirmatory assays. In the case of low copy samples close to the limit of detection, a cumulative curve with a much stronger signal is obtained with Duo CHIKV RT-qPCR assays compared to mono-target assays. This renders the technical validation of the assay truly easier; consequently, it reduces the number of inconclusive results that must imply to perform confirmation assays before final results, with increasing expenses and delayed results.

Thirdly, dual-target design can offer an improved sensitivity compared to mono-target assays. During acute infection with CHIKV, the observed levels of viremia are usually substantially higher than those observed with most other arboviruses, and specifically with flaviviruses [1,26,27,28]. Accordingly, although the question of sensitivity is less critical, we showed that the Duo assay sensitivity against strains representing the five main genetic lineages is at least equal and can be better than the sensitivity demonstrated by the single assays, individually.

## 5. Conclusions

As a conclusion, we demonstrated that combining two single systems into a dual-target assay do not impair sensitivity and specificity in CHIKV detection; moreover, it is a useful tool to face a potential emergency of CHIKV variants by newly evolving mutations. This study demonstrates that developing a Duo assay from two previously validated assays is easy and rapid. Thus, this study must be seen as a proof of concept that can be declined for other arboviruses and even other pathogens for providing a safe assay to be used routinely in clinical microbiology laboratories.

## Figures and Tables

**Table 1:** Primers and probes included in the Duo CHIKV RT-qPCR assay.

Reference	Primer/Probe	5′ → 3′ Sequence	Target	Position ^c^	Amplicon Size (nts)	Concentration
Pastorino et al. [9]	R-CHIK	CCAAATTGTCCYGGTCTTCCT	E1	10,568–10,588	208	900 nM
	F-CHIK	AAGCTYCGCGTCCTTTACCAAG		10,380–10,401		900 nM
	P-CHIK	FAM-CCAATGTCYTCMGCCTGGACACCTTT-TAMRA		10,479–10,504		200 nM
Panning et al. [10]	ChikAsI ^a^	GGCAAACGCAGTGGTACTTCCT	nsp1	295–316	82	600 nM
	ChikSI ^a^	TGATCCCGACTCAACCATCCT		234–254		600 nM
	ChikAsII ^b^	GGCAGACGCAGTGGTACTTCCT		295–316	77	600 nM
	ChikSII ^b^	CCGACTCAACCATCCTGGAT		239–258		600 nM
	ChikP ^$^	FAM-TCCGACATCATCCTCCTTGCTGGC-TAMRA		270–293		200 nM

^a^ primer designed based on sequences of the five lineages; ^b^ primer designed from sequences of Indian Ocean strains; ^c^ refers to the sequence of Ross strain of CHIKV (ECSA1 lineage) (001v-EVA1455; GenBank acc no MG280943); nsp, non-structural protein; E1, envelope glycoprotein 1. ^$^, TAMRA quencher replaces the BHQ1 of the original study.

**Table 2:** Strains tested to assess specificity of Duo Chikungunya virus (CHIKV) RT-qPCR assay.

Genus	Virus Species and Acronyms		Strain (Viral Load TCID_50_/mL)	Reference on EVAg or NCPV Catalog
*Alphavirus*	Chikungunya virus	CHIKV-West African	UVE/CHIKV/1983/SN/WA 37997 (10 ^6.22^)	001V-02448 (EVAg)
		CHIKV-Asian	H20235/STMARTIN/2013 (10 ^5.57^)	001N-EVAg1583 (EVAg)
		CHIKV-ECSA1	UVE/CHIKV/UNK/XX/ROSS (10 ^6.49^)	001v-EVA1455 (EVAg)
		CHIKV-ECSA2	UVE/CHIKV/2011/CD/Brazza_MRS1 (10 ^6.57^)	001v-EVA960 (EVAg)
		CHIKV-ECSA3	UVE/CHIKV/2006/RE/LR2006_OPY1 (10 ^8.49^)	001v-EVA83 (EVAg)
	Mayaro virus	MAYV	UVE/MAYV/1954/TT/TC625 (10 ^8.82^)	001v-EVA502 (EVAg)
	O’nyong-nyong virus	ONNV	UVE/ONNV/UNK/SN/Dakar 234 (10 ^4.22^)	001v-EVA1044 (EVAg)
	Semliki Forest virus	SFV	UVE/SFV/UNK/XX/1745 (10 ^4.42^)	001V-02468 (EVAg)
	Ross River virus	RRV	0005281v (10 ^8.16^)	0005281v (NCPV)
	Sindbis virus	SINV	UVE/SINV/UNK/EG/Egypt 339 (10 ^4.32^)	001V-02469 (EVAg)
	Western equine encephalitis virus	WEEV	UVE/WEEV/UNK/XX/47a (10 ^8.32^)	001v-EVA1479 (EVAg)
	Venezuelan equine encephalitis virus	VEEV	UVE/VEEV/UNK/XX/TC83 vaccine (10 ^9.42^)	001v-EVA1459 (EVAg)
	Eastern equine encephalitis virus	EEEV	UVE/EEEV/1999/XX/H178_99 (10 ^7.82^)	001v-EVA1480 (EVAg)
*Flavivirus*	Zika virus	ZIKV	UVE/ZIKV/1947/UG/MR766 (10 ^4.32^)	001v-EVA143 (EVAg)
	Dengue virus-1	DENV-1	UVE/DENV-1/2013/NC/CNR_17132 (10 ^7.57^)	001V-03151 (EVAg)
	Dengue virus-2	DENV-2	UVE/DENV-2/1996/PF/Papeete 341.175 (10 ^7.82^)	001V-03178 (EVAg)
	Dengue virus-3	DENV-3	UVE/DENV-3/UNK/RE/CNR_14448 (10 ^7.22^)	001V-03346 (EVAg)
	Dengue virus-4	DENV-4	UVE/DENV-4/2012/GF/CNR_16008 (10 ^8.49^)	001V-03366 (EVAg)
	Japanese encephalitis virus	JEV	UVE/JEV/2009/LA/CNS769 (10 ^5.57^)	001V-02217(EVAg)
	West Nile virus	WNV	UVE/WNV/2008/US/R94224 (10 ^7.32^)	001V-02224 (EVAg)
	Tick-borne encephalitis virus	TBEV	UVE/TBEV/2013/FR/32.11 WT-PCR (10 ^8.82^)	001V-02355 (EVAg)
	Yellow fever virus	YFV	UVE/YFV/UNK/XX/Vaccine strain 17D (10 ^6.32^)	001v-EVA67 (EVAg)
	Saint Louis encephalitis virus	SLEV	UVE/SLEV/UNK/US/MSI-7 (10 ^4.82^)	001v-EVA128 (EVAg)
	Usutu virus	USUV	UVE/USUV/1959/ZA/SAAR-1776 (10 ^5.32^)	001v-EVA138 (EVAg)
*Phlebovirus*	Toscana virus	TOSV	UVE/TOSV/2013/FR/113 (10 ^7.42^)	001V-02461 (EVAg)
	Rift Valley fever virus	RVFV	UVE/RVFV/UNK/XX/Smithburn vaccine (10 ^7.32^)	001V-02385 (EVAg)
	Sandly fever Sicilian virus	SFSV	UVE/SFSV/1943/IT/Sabin (10 ^6.82^)	001v-EVA77 (EVAg)

**Table 3:** Analytical sensitivity of Duo CHIKV RT-qPCR, Pastorino and Panning assays.

	**West African**	**Duo**		**Pastorino**		**Panning**	
Dilution	Viral RNA copies/µL	Positive/tested	Ct, Mean (SD)	Positive/tested	Ct, Mean (SD)	Positive/tested	Ct, Mean (SD)
E-6	603	6/6	32.1 (0.1)	6/6	32.6 (0.2)	6/6	31.8 (0.2)
2E-7	106	6/6	34.3 (0.25)	6/6	35.3 (0.2)	6/6	34.2 (0.1)
4E-8	24	6/6	36.5 (0.43)	6/6	37.3 (0.2)	6/6	36.4 (0.4)
8E-9	6	5/6	38.3 (0.36)	1/6	39.2	4/6	38.9 (0.2)
1.6E-9	-	0/6	-	0/6	-	0/6	-
3.2E-10	-	0/6	-	0/6	-	0/6	-
	**Asian**	**Duo**		**Pastorino**		**Panning**	
Dilution	Viral RNA copies/µL	Positive/tested	Ct, Mean (SD)	Positive/tested	Ct, Mean (SD)	Positive/tested	Ct, Mean (SD)
E-6	547	6/6	32.2 (0.2)	6/6	32.3 (0.1)	6/6	31.8 (0.2)
2E-7	136	6/6	34.3 (0.3)	6/6	34.8 (0.3)	6/6	33.7 (0.1)
4E-8	32	6/6	36.1 (0.2)	6/6	37.8 (1.1)	6/6	36.5 (0.8)
8E-9	8	6/6	38 (0.5)	4/6	38.5 (1)	2/6	38.6 (0.9)
1.6E-9	2	0/6	-	3/6	39.5 (0.3)	0/6	-
3.2E-10	-	0/6	-	0/6	-	0/6	-
	**ECSA1**	**Duo**		**Pastorino**		**Panning**	
Dilution	Viral RNA copies/µL	Positive/tested	Ct, Mean (SD)	Positive/tested	Ct, Mean (SD)	Positive/tested	Ct, Mean (SD)
E-6	146	6/6	34. 5 (0.2)	6/6	35 (0.2)	6/6	34.6 (0.5)
2E-7	39	6/6	36.4 (0.4)	6/6	37.5 (0.3)	6/6	36.8 (0.1)
4E-8	11	4/6	37.8 (0.6)	4/6	37.6 (0.5)	1/6	39.1
8E-9	5	1/6	39.2	1/6	38.7	0/6	-
1.6E-9	-	0/6	-	0/6	-	0/6	-
3.2E-10	-	0/6	-	0/6	-	0/6	-
	**ECSA2**	**Duo**		**Pastorino**		**Panning**	
Dilution	Viral RNA copies/µL	Positive/tested	Ct, Mean (SD)	Positive/tested	Ct, Mean (SD)	Positive/tested	Ct, Mean (SD)
E-6	859	6/6	31.9 (0.2)	6/6	31.2 (0.2)	6/6	31.4 (0.1)
2E-7	172	6/6	34.2 (0.1)	6/6	33.8 (0.5)	6/6	33.9 (0.3)
4E-8	44	6/6	36.2 (0.2)	5*/5	36.8 (0.7)	6/6	36.4 (0.8)
8E-9	13	6/6	38 (0.3)	1/6	37.4	4/6	38.4 (0.6)
1.6E-9	6	2/6	39.1 (0.03)	0/6	-	0/6	-
3.2E-10	-	0/6	-	0/6	-	0/6	-
	**ECSA3**	**Duo**		**Pastorino**		**Panning**	
Dilution	Viral RNA copies/µL	Positive/tested	Ct, Mean (SD)	Positive/tested	Ct, Mean (SD)	Positive/tested	Ct, Mean (SD)
E-6	875	6/6	31.4 (0.1)	6/6	29.7 (0.1)	5*/5	31.7 (0.2)
2E-7	204	6/6	33.6 (0.2)	6/6	32.1 (0.2)	6/6	34 (0.3)
4E-8	41	6/6	36.1 (0.2)	6/6	34.8 (0.5)	6/6	35.9 (0.4)
8E-9	13	5/6	38 (0.9)	5/6	37.1 (0.7)	5/6	38.3 (0.8)
1.6E-9	5	1/6	39.5	2/6	39.2 (0.03)	1/6	39,3
3.2E-10	-	0/6	-	0/6	-	0/6	-

* one replicate was removed due to a manufacturing error. Pastorino corresponds to Reference [9] (3’UTR), Panning correspond to Reference [10] (nsp1).
